# Building Resilient Cities: Climate Change and Health Interlinkages in the Planning of Public Spaces

**DOI:** 10.3390/ijerph19031355

**Published:** 2022-01-26

**Authors:** Eleonora Orsetti, Nicola Tollin, Martin Lehmann, Vanessa Agudelo Valderrama, Jordi Morató

**Affiliations:** 1 Department of Technology and Innovation, University of Southern Denmark, 5230 Odense, Denmark; nto@iti.sdu.dk; 2 Department of Planning, Aalborg University, 9000 Aalborg, Denmark; martinl@plan.aau.dk; 3 Càtedra UNESCO de Sostenibilitat, Universitat Politècnica de Catalunya—BarcelonaTech, The School of Industrial, Aerospace and Audiovisual Engineering of Terrassa, ESEIAAT, C/Colom, 08222 Terrassa, Spain; vanessa.agudelo@upc.edu (V.A.V.); jordi.morato@upc.edu (J.M.)

**Keywords:** climate change, health, city, public space, urban resilience, vulnerability, inequality

## Abstract

Greenhouse gases emissions resulting from the combustion of fossil fuels are worsening air quality and affecting the climate system. While climate change impacts on meteorological variables affects air quality by altering the concentration and distribution of pollutants, air pollution significantly influences the climate, leading to negative impacts on human health. Due to the combination of high temperatures, air pollution, and high population density, cities are particularly vulnerable to climate change impacts. The planning and design of public spaces aimed at climate change mitigation and adaptation can result in multiple co-benefits for human health, while reducing social inequalities. To address the major research gaps in the communication between health and planning experts, and the lack of capacity among public sectors and policy makers, it is necessary to promote capacity building and knowledge sharing between the planning and health sectors. The purpose of this article is to develop preliminary recommendations for a process that allows a comprehensive assessment of the interlinkages between climate and health, social, environmental, and economic vulnerabilities, and the quality of the urban spaces, to support local governments, policymakers, and education institutions in making informed decisions for public spaces. The methods applied were a literature review and interviews with experts.

## 1. Introduction

Greenhouse gases (GHGs) emissions resulting from the combustion of fossil fuels are worsening air quality and with high confidence affecting the climate system, inducing changes at an unprecedented rate [1,2]. 

Air pollution significantly impacts human health and, according to the European Commission, it is perceived as the second biggest environmental concern for Europeans, after climate change [3]. Exposure to particulate matter (PM2.5) is a major cause of premature death and illness, causing around 400,000 premature deaths per year in Europe [3]. Although air pollution affects the health of the whole population, some population groups are more vulnerable, such as children, the elderly, pregnant women, persons with disabilities, indigenous people, homeless, refugees, migrants, lower socio-economic groups, and people with pre-existing health problems [3].

Climate-change-induced effects, such as higher temperatures and humidity, can influence the frequency and intensity of extreme events, the spread of vector-borne diseases, increase the intensity of heat waves and contribute to sea-level rise, with consequent direct and indirect impacts on human health and well-being [1,4].

Air pollution and climate change are thus interconnected: while the effects of climate change on meteorological variables contribute to the deterioration of air quality by altering the concentration and distribution of pollutants, the latter significantly influence the climate system, leading to more negative impacts on human health [4,5,6].

Tapia et al. [7] argue that climate-driven risk is determined not only by hazard and exposure but also by vulnerability, as defined by the United Nations Office for Disaster Risk Reduction [8]. The health vulnerability to climate change is influenced by different components: the sensitivity of social systems to changes in weather and climate, dependent on the demographic structure, the exposure to climate-related hazards, and the adaptation strategies adopted to reduce negative health outcomes [9]. Furthermore, the socio-economic status can influence the accessibility to green public spaces, increasing environmental inequality [10]. Due to the combination of these factors, lower socio-economic groups in urban areas are disproportionately vulnerable to air pollution, noise, and high temperatures [10]. 

Physical, political, social, and economic conditions can thus contribute to increasing both health inequality, defined by the World Health Organization as the difference in the distribution of health determinants across the population caused by biological variation, and health inequity, which describes the variation attributable to the uneven distribution of causes and outcomes of poor health [11,12]. In the short-to-medium term, therefore, climate change impacts on human health will be determined mainly by populations’ vulnerability, resilience, and adaptation actions. In the long term, the impacts will depend on the actions that are now taken to reduce emissions and avoid exceeding temperature thresholds and irreversible tipping points [13]. A temperature increase of 1.5 °C, and even more of 2 °C, will worsen social, demographic and environmental inequalities [2,14,15].

Cities are at the same time one of the largest contributors to GHGs emissions and are particularly affected by the impacts of climate change due to the combination of high temperatures, air pollution, and high population density [16]. Climate change affects public health in urban areas also due to the greater frequency of extreme weather events, which increase the risk of mortality and morbidity and lead to the disruption of infrastructure and services [17]. Climate projections show an increment in the frequency, duration and intensity of heat waves, which, enhanced by the urban heat island effect, represent one of the deadliest climatic extremes in urban areas, accounting for an estimated 70,000 premature deaths in 2003 in Europe [18,19].

The urban form, the building configuration, and the presence of vegetation can play a decisive role in the reduction, prevention, or intensification of climate change impacts in cities [20]. For example, heavy reliance on private transport systems typical of sprawl cities could increase the levels of air pollution and traffic accidents, while reducing physical activity [20]. Consequently, depending on the urban structure, planning strategies, existing geomorphological factors, vulnerability and risk exposure, some areas may experience climatic conditions worse than others [21]. As the quality of urban space is, therefore, not homogeneous throughout the city, special attention must be paid to the uneven distribution of environmental benefits and disadvantages, in order to avoid forms of climate injustice [21]. Understanding the risk factors in a city can help to find preventive solutions, investigating social and economic variables, the presence, distribution, and accessibility of green areas, including public ones, and the existing health conditions [22]. Assessing climate-related risks and how health systems can address them is thus one of the critical first steps that governments must take to improve population health, a fundamental element of urban resilience [13,23].

Urban resilience is defined by the United Nations Human Settlement Program (UN-Habitat) as the capacity of an urban system to respond to and absorb shocks, transforming and adapting in the perspective of a sustainable development [23].

Meerow et al. further argue that, as climate change and urbanization are likely to intensify the already unstable nature of cities, urban resilience must operate in a state of non-equilibrium, with the aim of maintaining or quickly restoring desired functions [24]. Because of the dynamic and complex nature of urban systems, a return to a prior condition after a disturbance is highly improbable [24].

Urban resilience can be operationalized through a range of tools and indexes that enable the assessment of the resilience of cities to different shocks and challenges: several international public and private actors have developed these tools, such as the United Nations Office for Disaster Risk Reduction (UNDRR), the World Bank, UN-Habitat, and the Rockefeller Foundation, with the aim of supporting city governments in developing local plans and strategies [23]. Ensuring good health for citizens is therefore crucial to building resilient and thriving communities, fostering social interaction and supporting vulnerable populations, and it is one of the most effective indicators of sustainable development [25]. Healthy cities are sustainable and resilient [25]. Although health is a critical dimension of the resilience of cities, resilience strategies often do not address opportunities to improve the health of the population through urban planning policies and actions [23]. On the contrary, urban planning that integrates health issues offers the opportunity to improve urban health and reduce social inequalities, advancing the achievement of the Sustainable Development Goals [25].

The SDG 11 “Make cities inclusive, safe, resilient and sustainable” includes the social, economic, and environmental dimensions, and focuses specifically on the urban context [26]. In particular, Target 11.7 highlights the importance of providing green public spaces, safe, inclusive and accessible for all [27].

Since the 1960s, public spaces experienced substantial disinvestments from governments funding, with an increasing role of private actors in the development and management of the public realm; the quality of public spaces consequentially decreased, often in proportion to the distance from the city center [28]. As privatization is profit driven, public access remains at the discretion of the owner and can be refused to any person or activity that could be seen as potentially disruptive [28]. Public spaces can increase social ties by providing opportunities to meet, see, and hear people while sharing the same space, therefore their privatization can lead to the deterioration of community cohesion, decreasing the level of social support for vulnerable individuals [29]. Hence, while the unequal distribution, quality, location, and access to public spaces directly impacts human health, the provision of a network of accessible, inclusive, and green public spaces supports health equity and give the freedom to all the citizens to enjoy them [30]. Public spaces should thus ensure both access and accessibility, the former by giving the opportunity to enter the space and the latter by supporting equal access and usage for people with disabilities and different social groups [11]. 

In “Life between buildings. Using public space”, Jan Gehl argues that the quality of outdoor areas influences the nature of the ongoing activities. In poor quality spaces, only necessary activities occur; on the contrary, high-quality spaces attract people to sit, eat, play, and relax, as the surrounding environment is perceived as attractive [31]. Public spaces are thus the center of social interaction, where we learn about ‘others’, and they play an important role in sustaining the public sphere [28]. Architects and planners, therefore, can increase the opportunities for people to meet and engage in social interactions [31].

This paper thus aims at understanding the interlinkages between climate change and health and the exposure to multiple stressors in public spaces not only according to urban form, infrastructure, and health system, but also to socio-economic systems and community cohesion.

### Introduction—Summary Analysis

- GHGs emissions are the cause of both deterioration of air quality and climate change.
- Air pollution and climate change have a reciprocal influence, leading to negative impacts on human health.
- Impacts on human health are determined mainly by exposure to climate-related hazards, populations’ vulnerability, resilience, and mitigation and adaptation actions.
- Cities are the largest contributors to GHGs emissions and at the same time extremely vulnerable to climate change impacts, especially urban lower socio-economic population groups.
- Assessing climate-related risks and the response of health systems are critical steps to improve population health and increase urban resilience.
- Urban planning strategies should aim at improving the health of the population and reduce social inequalities, advancing the achievement of the Sustainable Development Goals.

## 2. Materials and Methods

### 2.1. Problem Formulation

Research gaps have been found in the definition of the role of social, environmental, and economic inequalities in magnifying exposure to environmental risk, influencing sensitivity and access to green public spaces, and increasing vulnerability to climate change [29,31].

While the research on climate risks is relatively developed, knowledge on the social factors that drive individual or community health vulnerability to climate change is limited [32]. Difficulties may arise in the collection and analysis of health-specific qualitative and quantitative data as a result of the complex understanding of the determinants of health in urban areas [33]. Furthermore, the communication gaps between urban planners and the health sectors could be a major challenge in assessing the risk posed by climate change to the health of urban residents [17].

Current methods for assessing the direct costs and effects of climate adaptation strategies usually do not address health co-benefits and risks [34]. The United Nations Environment Program (UNEP) states that the better integration of health into climate planning, improved capacity of health systems, development of policies aimed at reducing climate vulnerability of the built environment, and further implementation of early warning systems are essential to building local health adaptive capacity [35].

A great number of evaluation processes and strategies have been found in the literature, either focusing on climate impacts, health status, urban space quality, or vulnerabilities. However, a limited number explores the role of inequalities in affecting the interlinkages between climate change and health, and how these can be addressed through public space planning strategies. Therefore, the purpose of this article is, firstly, to investigate how public spaces can contribute to addressing the causes and effects of climate change and reducing related health impacts while improving the quality of the built environment. Secondly, the paper aims to highlight key knowledge gaps and develop a preliminary set of recommendations to establish a functional process for making informed planning and design decisions, particularly by architecture and engineering professionals, aimed at reducing climate change impacts and protecting health, with specific reference to public spaces.

Based on the above considerations, the research question was structured as follows:

How to support the planning and design of healthy and inclusive public spaces, addressing both causes and effects of climate change, to build resilient and livable cities? 

The main research question was supported by the following sub-questions:

- How can planning strategies for public spaces contribute to climate change adaptation and the mitigation of and reduction in social and environmental inequalities, improving health in cities?
- How to assess the main challenges and research gaps related to the interlinkages between climate change and health, including considerations on vulnerabilities, in planning public spaces?
- What co-benefits can result from the planning of healthy and inclusive public spaces?

The paper is structured as follows: Section 2 provides the description of the research methods and the analytical framework, outlining the collection and analysis of the results, later detailed in Section 3. Finally, the discussion of the results and research gaps and the conclusion are presented in Section 4 and Section 5.

### 2.2. Analytical Framework

The planning and design of resilient, healthy, and inclusive public spaces in an era of climate change need to have a strong focus on health and the public good [22].

During the research and analysis phase, a normative approach was used to understand how to strengthen the system of public spaces, especially in relation to health co-benefits. The notion of health as a state of physical well-being associated in cities with social, economic, and environmental determinants supports the idea that it is a public good influenced by adaptive capacity and self-management [17].

According to the Charter of Public Space, the contribution of the Biennial of Public Space in Rome (2013) in collaboration with the United Nations Program on Human Settlements (UN-Habitat), the city can be considered as a public good, as the physical and symbolic space of expression for all [36]. The Charter defines public spaces as “[…] all spaces that are publicly owned or of public use, accessible to all for free and without profit motive, including streets, local public markets, parks, public squares, beaches, recreation areas, plazas and other publicly owned and managed outdoor spaces” [36] (p. 11). As the public spaces contribute to public health, the resource management approach of these commons should thus arise from a participatory and inclusive process in the analysis, design, planning, and management phases [37]. Places can thus be regarded as complex, interconnected socio-spatial systems susceptible to changes over time [38]. In the context of climate change and health, planners can act as facilitators in the planning processes, where collaboration among different stakeholders takes place, in order to create a positive working environment and maximize participation [39].

The milestone for understanding the interconnections between urban planning, design, governance, and their related impacts on human health was set by the approval of the 2030 Sustainable Development Agenda and the definition of the 17 Sustainable Development Goals (SDGs) [40]. Moreover, health emerged as a cross-cutting issue with the adoption of the New Urban Agenda at Habitat III, the United Nations Conference on Housing and Sustainable Urban Development in 2016, and was identified as a core component of urban planning [40].

The SDGs provide a framework for addressing the linkages between the global temperature increasing of 1.5 °C or 2 °C and the goals of ending poverty, protecting the planet, and ensuring that all people enjoy peace, health, and prosperity while promoting social justice and equity [14]. The 17 SGDs and the 169 targets are integrated and indivisible and support the 3 dimensions of sustainable development: economic, social, and environmental [27]. The interaction between climate and the SDGs is a growing process in the scientific literature and its understanding is a crucial point in selecting appropriate mitigation measures and promoting sustainable development [41].

**Figure 1:**
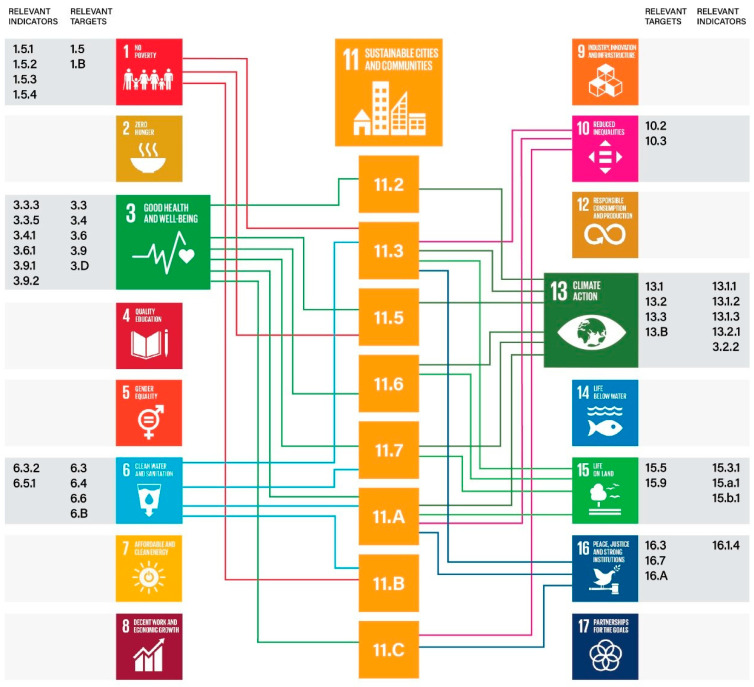
Sustainable development goals (SDGs), targets and indicators in connection with SDG 11. Adapted from: United Nations Human Settlement Program (UN-Habitat). SDG 11 Synthesis Report: Tracking Progress towards Inclusive, Safe, Resilient and Sustainable Cities and Human Settlements. 2018.

The achievement of the SDGs is also advocated by the Paris Agreement, which supports long-term sustainable development and addresses the health risks associated with climate change through mitigation and adaptation [42]. The SDGs can thus be applied as an operational framework for considering urbanization at the global level, linking Goal 11, described in Section 1 as central in this research, with targets and indicators from the other SDGs. Goals, targets, and indicators are interconnected and dependent on each other, since contributions from sectors different from health also serve as instruments for achieving progress towards healthy cities and communities, as, for example, the relationship between mental health and non-communicable diseases and the environmental and socio-economic determinants [40]. Figure 1 shows the interlinkages between SDG 11 and other SDGs’ targets and indicators.

The selection of relevant targets and indicators showed in Figure 1 was based on their relations with the targets of Goal 11, according to their different focus. Environmental components relative to air and water quality, water sanitation, vector-borne diseases and transports (targets 3.3, 3.6, 3.9, 6.3, 6.4, 6.5, 6.6, 6B and relative indicators) are connected with the targets of Goal 11 focused on urbanization (11.2, 11.3), climate change mitigation and adaptation and resource efficiency (11.B), and air and water quality (11.6).

Targets relative to capacity building (16.A), climate change mitigation and adaptation (13.1, 13.2, 13.3, 13.B and relative indicators), risk reduction (3.D, 15.3 and relative indicators), urban and environmental planning and biodiversity protection (15.5, 15.9, 15.A, 15.B and relative indicators), and participatory decision making (16.7) are connected with the targets of Goal 11 focused on sustainable urban planning (11.3, 11.A, 11.C) and public spaces (11.7). The target 11.7, particularly relevant in this paper, and target 11.5 are directly connected with targets focused on social, political and economic inclusion and reduction in inequalities (10.2, 10.3 and relative indicators), reduction in vulnerabilities (1.5, 1.B and relative indicators), mental health (3.4), and reduction in injustice (16.1, 16.3 and relative indicators). The SDGs served as the basis for analyzing the connections of the data retrieved from the literature and the interviews.

The conceptual framework presented in this article thus links the SDGs and urban resilience by focusing on urban health. The analysis of data, collected through a literature review and semi-structured interviews with stakeholders, aimed at identifying health as a determinant, outcome, and indicator of sustainable development and urban resilience, as presented in Section 3.

Based on the themes outlined in the analytical framework, thematic analysis and secondary data analysis were used in this research to investigate possible connections and patterns between themes and concepts discussed in the interviews and the literature [43].

### 2.3. Methods

Based on the framework and the research gaps presented in Section 1, the method of the literature review was employed to provide the state of the art on the integration of climate and health issues into public space planning strategies, as well as the existing assessment tools, supported by semi-structured experts’ interviews. Since the subject of the study involves various areas, multidisciplinary research covering engineering, environmental, and medical fields was considered appropriate.

The limitations of the current research include its focus on the urban context of the global north. Because of the complexity of the topic, the impacts of the COVID-19 pandemic were excluded in the data collection and analysis, and it focused solely on the interlinkages between climate change and health in cities. In addition, in the analysis of the evaluation systems, priority was given to those focused on the urban context, while those that focus on health and climate change on a general or national level were excluded. The research was limited to considerations regarding outdoor public spaces, excluding for the moment the analysis of indoor spaces features and challenges.

#### 2.3.1. Literature Review

A literature review was conducted to outline the key knowledge gaps and the areas where further research is needed, which made it possible to identify theories and concepts related to climate, health, planning, and public spaces and their relationship.

A search based on the following keywords was conducted on the scientific databases Scopus and Web of Science among articles, reviews, and book chapters in English, without temporary restrictions: “climate change”, “health”, “urban health”, “cit*”, “public space*”, “vulnerabil*”, and “urban”. Secondly, the literature containing the terms “indoor”, “forest”, “food”, “waste”, and “hospital*” was excluded.

An analysis of the relevant literature retrieved outside the scientific spectrum was considered necessary, due to its significant contribution to a review [44]. By including a wide range of sources, such as academic papers, committee and government reports, and conference papers, the comprehensiveness of the study is increased and might lower publication bias [44]. In this paper, it was considered relevant to review the contributions of the main international organizations dealing with climate and health in the urban context, e.g., World Health Organization (WHO), UN-Habitat, European Environment Agency (EEA), and selected architecture, engineering and consulting firms, e.g., Henning Larsen, Gehl, and Rambøll.

#### 2.3.2. Interviews

Interviews with experts were employed as a complementary method to provide new contributions or insightful perspectives on the findings of the literature search [45]. Expert insight can provide an overview of relevant knowledge in the field and offer solutions based on practical experience and strategies [46]. Therefore, semi-structured qualitative interviews were preferred, as they tend to be less structured than interviews used in quantitative methods. Appropriate topic guidance framed the data collection, avoiding closed-ended questions and providing opportunities for respondents to step outside the interviewer’s guidance in order to identify their perspectives, additional issues, or unanticipated directions [47]. The formulations of the questions were related to the institutions involved and did not aim at a personal opinion of the respondents [47]. 

Five interviews were conducted by the corresponding author with seven experts and practitioners at three levels. Experts from the European Environment Agency (EEA) and United Nations Human Settlement Program (UN-Habitat) were interviewed in order to understand the main theories and guidelines on the research subjects, and the main assessment tools and strategies.

Aleksandra Kazmierczak, Program HSR1—Air Pollution, Environment and Health at EEA, author of several publications and guest lectures on health and climate vulnerability, public spaces, and just adaptation, was interviewed on 19 April 2021. The interview focused on the role of public spaces in relation to climate variables and health, the main strategies aimed at just adaptation, and the factors that influence health and climate vulnerability and had a duration of 33 min.

Joy Mutai, Associate Coordination Officer at UN-Habitat, author of several publications on public space and city-wide assessment tools of UN-Habitat, and Pamela Carbajal Perez, Urban health and regional planner consultant at UN-Habitat, author of several publications on health assessment tools of UN-Habitat and the World Health Organization (WHO), were interviewed on 30 April 2021. The interview was conducted as an open dialogue about tools and strategies to assess and support urban planning and design strategies for public spaces in relation to health and climate. The duration was 36 min.

The interview held on 20 April 2021 with the urban planning expert Camilla Van Deurs, City Architect of Copenhagen and former Partner and Director of Gehl Architects, was aimed at understanding how urban strategies for public spaces, climate change mitigation and adaptation, and health issues are managed in the city of Copenhagen, to obtain a deeper insight into decision-making processes and the evaluation and data collection strategies in support of the design phase. The interview lasted 39 min and was aimed at understanding the main strategies of the Copenhagen municipality regarding the impacts of climate change on urban health, with a focus on public spaces.

Within the professional firms, the architecture offices Gehl Architects and Henning Larsen were identified as the leading companies in relation to the design of public spaces and the evaluation of social interactions.

Krister Jens, Industrial PhD Fellow at Henning Larsen and DTU, in charge of a project on data-driven knowledge, was interviewed on 28 April 2021. The addressed topics were the social and economic impact of climate change on health and the strategies aimed at creating value and attracting private investors in public projects. The duration of the interview was 33 min.

The interview with Sophia Schuff, Project Manager at Gehl, expert in healthy urban design approaches and tools, and Alexander Spitzer, Associate at Gehl and project manager of the Public Space and Public Life Digital Platform, was held on 6 May 2021. The interview had a duration of was 41 min and focused on social and economic impact of climate change on health and the strategies aimed at creating value and attracting private investors in public projects.

Two of the interviews involved two experts, one with a health perspective and one with an urban focus. Experts had equal levels of hierarchy within their organization, with no power relationships among them that would prevent unprompted responses.

The comparability of the responses given by the experts was facilitated by the common framework provided by the interview guide: a thematic guideline was established, which guided the dialogue in a flexible way, without preventing the respondents from expanding their answers; the questions aimed at understanding general principles and responses at a supra-personal level of the respondents’ knowledge, related to their respective organizations [47]. 

## 3. Results

The following sub-sections are structured to answer the research questions presented in Section 2.1. The results were analyzed according to the analytical framework presented in Section 2.2.

### 3.1. Role of Planning Strategies in Tackling Interlinkages between Climate Change and Health

Sub question 1: How can planning strategies for public spaces contribute to climate change adaptation and mitigation and the reduction in social and environmental inequalities, improving health in cities?

The New Urban Agenda emphasizes how open, safe, inclusive, well-connected, and distributed public spaces improve resilience to disasters and climate change impacts while promoting physical and mental health [36]. 

The equal distribution of public spaces can reduce economic and social segregation, where people from all social classes can meet and engage in spaces that fulfil the needs of the residents, considering that not everyone has the same needs, or the same needs at the same time [28]. By building a community sense and facilitating economic development, promoting culture, and empowering civic identity, public spaces function as arenas for social interaction and drivers for sustainable development, promoting social inclusion, safety, reduction in violence, and gender equality [36]. The provision of green spaces contribute to better air quality, noise and heat island effect reduction, and promote biodiversity [36]. Health benefits are especially relevant where residents experience health inequalities, as green spaces encourage physical activity and reduce stress while contributing to climate mitigation and adaptation [48]. Furthermore, the economic disparities between the lowest and highest income groups are minor among those living in the greenest areas than in the least green areas [48]. Hence, assuring a high level of access to green public spaces through urban planning policies, and focusing on the social dimension of the adaptation strategies, can strengthen social cohesion and reduce health inequity [10]. The provision and maintenance of green public spaces are thus an integral part of safe and sustainable cities’ strategies [36,49]. 

Since the EU policies rarely tackle the social distribution of environmental risk and the benefits brought about by high-quality environments, the first action is to integrate these goals into policies [10]. Governments should also integrate health, equity and environmental considerations into urban planning policies and practice, including cost–benefit analysis, to ensure future-proof cities [13].

Well-planned cities are more resilient to the impacts of climate change than unplanned ones [39]. The presence of high-quality public spaces is representative of a city’s quality of life, while prioritizing walking and cycling can contribute to the reduction in GHGs emissions; in this regard, Copenhagen is one of the leading cities in the world aiming at becoming carbon neutral in 2025, also by raising the percentage of travel by walking, cycling, or public transport to 75% [39]. As outlined by City Architect of Copenhagen Camilla Van Deurs, the management of public spaces is never a simple issue related just to climate change, health, biodiversity, or GHGs emissions reduction, but rather a combination of those issues through urban planning. For example, the implementation of a health hub in the city enabled the integration of both outdoor and indoor physical activities into an overall city plan, increasing the health and well-being outcomes (Van Deurs, 2021)]. Providing a high level of soft-traffic connectivity between the city’s public spaces can ensure that people have access to various green and recreational open spaces also outside their districts, encouraging people to travel between them [28] (Van Deurs, 2021). Urban planning that promotes health should, therefore, provide cycling infrastructures to connect different spaces, access to healthy food, access to green areas and nature, including biodiversity, and climate change mitigation and adaptation [30]. Furthermore, to act on social structures, the direct engagement of the community should be part of the process since the beginning, increasing the sense of belonging, inclusion, and trust [11].

Evaluating the quality of urban space, accessibility, access, use and users, and safety aspects may support the understanding of different forms of inequality and determine whether a project can improve community health outcomes [11].

The need for a broader perspective on climate change adaptation in cities was expressed also by the EEA expert’s understanding of “Just Resilience”, a concept that includes a comprehension of the different cases of procedural justice in terms of climate impacts and their distribution across society (Kazmierczak, 2021). This supports the understanding of where greater public funds are needed, for a fair distribution of resources, to benefit the most vulnerable ( Kazmierczak, 2021).

As mentioned in Section 1, air pollution and climate change are interconnected [3]. As greenhouse gases and air pollutants share the primary sources of emissions, policies aimed at limiting the emissions of one can potentially benefit the other; mitigating the effects of air pollution can therefore also lower climate change impacts, with co-benefits for human health and the environment [3].

The first Intergovernmental Panel on Climate Change (IPCC) Report and the United Nations Framework Convention on Climate Change recognize the importance of social justice, concerning the unequal distribution of climate impacts within generations (inter-generational equity) and between countries and social groups (intra-generational equity) [32].

The social impact of climate change often presents a knowledge gap for urban practitioners, including planners, who usually manage environmental projects without integrating health competencies [50]. Vice versa, local health authorities usually lack the professional capacity for physical and spatial planning [39]. UN-Habitat experts confirmed the common lack of expertise and communication between the health and planning sectors, and the urgency of linking them to integrate health data into urban strategies, for example, by mapping obesity and non-communicable diseases (Carbajal & Mutai, 2021). Including health stakeholders in the planning process might facilitate the health-promoting design of public spaces and support urban decision-makers to achieve the goals of the New Urban Agenda, by assessing the health and equity impacts of urban policies, as well as their benefits and costs [25]. The monitoring of health impacts in public spaces needs to be implemented, as was accomplished in the car-free air pollution strategy in Nairobi, where the monitoring of impacts demonstrated improved air quality and greater use of space by the community (Carbajal & Mutai, 2021).

Furthermore, databases, such as the WHO Global Health Observatory, which includes indicators on health outcomes, can be used to monitor urban policies by linking them to SDGs indicators [25] and potentially offer further guidance for planners. Sharing knowledge is, therefore, crucial, also through the different stages of the planning process. To this end, the goal of the European Commission, the EEA, and partner organizations is to create a connection between health and climate change mitigation and adaptation expertise through the European Climate and Health Observatory and the database Climate-ADAPT, which includes documents on adaptation at the local level [32] (Kazmierczak, 2021).

The main adaptation strategies to address health risks from climate change have been identified to establish hot-weather responses and early warning systems, improving disaster management programs, increasing health capacity, informing the population about air quality and GHGs emissions, identifying the vulnerable population, and increasing surveillance of water systems [34]. 

#### Role of Planning Strategies in Tackling Interlinkages between Climate Change and Health—Summary Analysis

- The provision of a network of public spaces can improve urban resilience, reduce climate change impacts, and lower social and economic segregation and health inequalities.
- Green public spaces improve air quality, reduce the heat island effect and encourage physical activity.
- Urban policies aimed at limiting GHGs emissions can generate co-benefits on health.
- The main knowledge gap was found in the lack of communication between the planning and health sectors.
- The integration of health stakeholders in the planning process could reduce climate-change-related impacts on health.

### 3.2. Assessment Process

Sub question 2: How to assess the main challenges and research gaps related to the interlinkages between climate change and health, including considerations on vulnerabilities, in planning public spaces?

Vulnerability and Adaptation Assessments provide the evidence for health adaptation planning responsive to vulnerabilities and inequalities, supporting governments in formulating their response to climate risks. They also provide a framework for monitoring the health impacts of climate change, supporting capacity building, and advocating reasons for investing in health protection [13].

As outlined in Section 1, a number of research gaps led to an evaluation of which tools are available to identify climate and health risks, vulnerability and exposure, and planning processes and phases. During the analysis of the evaluation systems, the priority was given to those focused on the urban context, while the tools that focus on health and climate change on a general or national level were excluded.

UN-Habitat has developed several tools to analyze and assess vulnerability and climate change risks:

- “Climate Change Vulnerability and Risk” defines a methodology to analyze community and system exposure, sensitivity, and adaptive capacity founded on a participatory and community-based approach [51]. It enables the identification of low-risk areas for future development with the goal of informing participatory, community-driven adaptation planning processes, develop early warning systems, and build capacity [51].
- “Planning for climate change: a strategic, values-based approach for urban planners” offers a comprehensive overview of strategies to address climate change through urban planning processes at the local level, promotes inclusive participatory approaches, and supports the development of adaptive capacities for urban planners, stakeholders and professionals from related sectors, increasing communication and cooperation between different levels [39].
- “City Resilience Proofing Tool” enables the assessment of the level of social protection and services, in order to increase social resilience, social inclusion and reduce vulnerability to present and future impacts, implementing early warning systems for extreme events [17,52]. Mapping is useful to understand the geographic distribution of vulnerabilities and explain the results [53].
- “The City Resilience Profiling Tool (CRPT)”, based on the exposure analysis of current and projected climate data in combination with sensitivity data, allows to assess the degree of the biophysical impacts of climate change and the adaptive capacity of the city in relation to its social, institutional, and physical elements [54]. Collaborative stakeholder engagement combined with governance, urban development, and climate analysis expertise is essential to prioritize actions aimed at improving the resilience of vulnerable populations [54].

Other tools to assess health vulnerability and inequality have been developed:

- “Urban HEART: Urban Health Equity Assessment and Response Tool” (WHO) is a decision-support tool focused on understanding vulnerabilities and inequities, health outcomes and risks across different economic groups and promotes community participation in cross-sectoral collaborative actions, to drive political decisions and resource allocation towards health equity [55].
- The “Health Impact Assessment (HIA)” allows evaluating a policy, program, or project through a combination of methods and tools, to understand their potential health effects and how they are distributed across the population. Therefore, understanding population demographics is the foundation for identifying vulnerable population groups [56].
- “Inclusive Healthy Places—A Guide to Inclusion and Health in Public Space: Learning Globally to Transform Locally” guides professionals and communities in the creation of public spaces that support inclusion, health, and health equity. Inclusion can thus be understood not solely as an outcome, but also as a process that engages participants, increasing a sense of trust among them and enabling the achievement of a shared vision [11]. Multiple stakeholders should be involved, including planners, designers, and policymakers, as well as health professionals, community leaders and members [11].The framework can support professionals from different backgrounds (government, planning, design, and health) to collaborate in the promotion of health equity [11]. To this end, an understanding of demographic data is needed to identify gaps and barriers to good health and the drivers of social inequalities in health [11].

In the municipality of Copenhagen, the first step of urban design processes is to define the demographics of an area, in order to understand the users of the space and how the planned activities can reflect the needs of the community (Van Deurs, 2021). However, the top-down approach of demographic analysis must be combined with bottom-up engagement processes, since the first step in improving climate conditions is to understand people’s behavior, and then shape it towards healthier habits (Van Deurs, 2021). Hence, even in different situations and projects, the starting point should always focus on people (Van Deurs, 2021).

- The “Compendium of Inspiring Practices: Health edition” emphasizes the importance of collaboration between health professionals and urban planners, since their different approaches can stress the benefits of good urban planning practices for health, putting the latter at the center of the planning process and not just considering it as an outcome [33].
- In “Approach for Assessing Human Health Vulnerability and Public Health Interventions to Adapt to Climate Change”, Ebi at al. developed a method for assessing the potential impacts of climate change on human health, to support policymakers in making informed evidence-based decisions aimed at increasing resilience to current and projected climate impacts [9]. The current distribution of climate impacts on health, the existing strategies and measures to address them, and the estimate future health impacts resulting from climate or socioeconomic factors are analyzed to identify possible policies and adaptation measures to reduce climate-related health impacts and increase capacity, thereby improving resilience to climate change [9].

Urban spaces can increase the livability of a city by allowing people to experience positive physical and mental well-being, and to feel a sense of belonging to their place and community [57]. Multiple factors that contribute to the resilience of urban spaces can be assessed:

- “The City Resilience Framework”, developed by The Rockefeller Foundation and ARUP, combines the physical aspects of cities with the non-tangible aspects of human behavior, and aims to generate the dialogue and involvement of new actors within civil society, local government and business to facilitate the development of resilient cities [58]. It embraces 12 key objectives that outline the features of a resilient city, grouped into 4 categories: health & well-being, economy & society, infrastructure & environment, and leadership & strategy [58].
- “The City Resilience Action Planning (CityRAP)” tool (UN-Habitat) assists small and medium-sized cities, or districts in large cities, to strengthen their resilience through practical actions and collaboration between different stakeholders [59]. Bottom-up planning approaches provide the opportunity to engage stakeholders, city dwellers, and communities in mapping the risks [59].
- The UN-Habitat “City-Wide Public Space Assessment” tool enables to identify challenges in the development of long-term strategies for public places by assessing the provision of spaces, their accessibility, distribution, and connectivity, with the aim of assessing their quality and potential disparities and to develop a city-wide strategy [60].
- “Citywide public space inventory and assessment tool” (UN-Habitat) provides a flexible framework for assessing the quality and quantity of public spaces, their network, distribution and accessibility, and the level of social inclusion. The development of the strategy should involve multiple stakeholders, analysis of data on maps, and co-design strategies [61]. A monitoring and evaluation phase should be incorporated, followed by the implementation of selected priorities [61]. Each strategy should include an action plan, jointly agreed upon by all stakeholders, that can anticipate future needs [61].
- The tool “Public Space Site-specific Assessment” (UN-Habitat) evaluates the quality of public spaces according to five dimensions: accessibility, green spaces, comfort and safety, services, use and users [62]. It is based on the high level of participation of local authorities, experts and community members in order to understand where to allocate resources, create green and blue networks, support biodiversity, and provide climate change mitigation and adaptation strategies [62]. The result is a set of recommendations that may be utilized as input for urban design by architects and planners, in accordance with community needs [62]. An evaluation and implementation phase of the design solutions are required to assess whether they provided positive outcomes, improve the design and share knowledge [62]. The tool allows to assess the characteristics of the space within a 5 min walking distance, with the aim of planning the 15 min city [63].

Compact cities, such as the 15 min city, have proven to be capable of preventing disease and promoting health, improving social well-being, addressing climate change and environmental degradation, and supporting greater urban resilience in response to public health measures such as lockdowns [13]. This concept gained recently greater interest also among private developers and clients, who are expected to provide a diversity of uses, walkability, and accessibility to urban spaces (Schuff & Spitzer, 2021). 

#### 3.2.1. Assessment Process—Analysis of Tools

**Figure 2:**
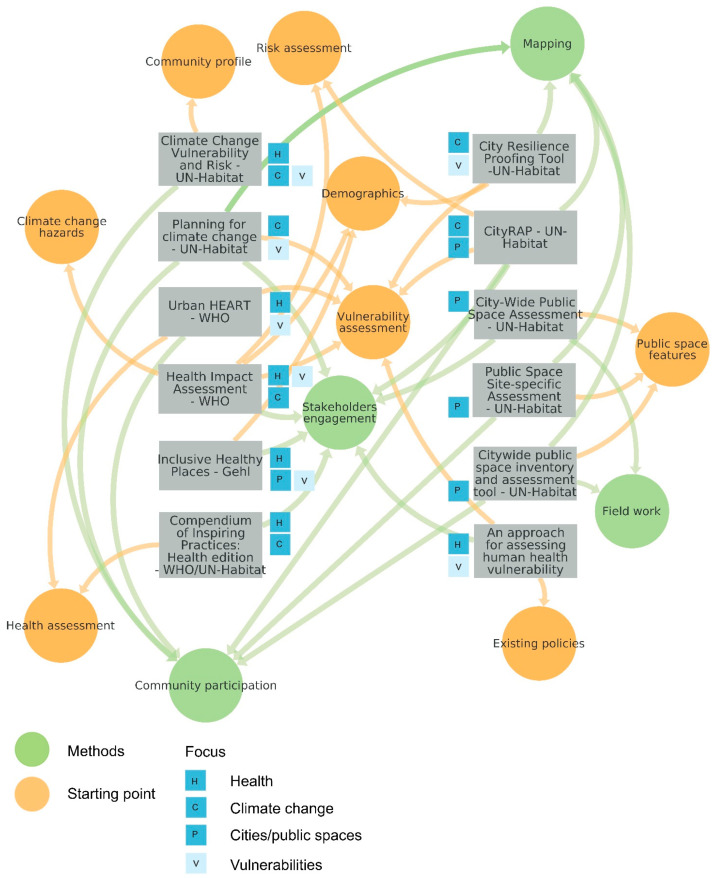
Assessment tools analysis (Source: Authors).

Main strains were found in the different tools and are summarized in Figure 2, to understand the common features, methods, and the focus of the assessment process.

The analysis of the tools showed in Figure 2 revealed that, although some of them consider the interrelations between health and climate in cities, the main subject of investigation is never a combination of the three aspects.

Common patterns can be identified in the initial point of the analysis (most often demographic data and vulnerability assessment) as well as the methods used. They include community participation, involvement of stakeholders from different sectors, and mapping and analysis of climate and health impacts, exposure, and vulnerability. The analyzed tools emphasize the importance of cross-sector collaboration and a thoughtful planning process, which must include monitoring and implementation phases.

Breaking down siloed knowledge between different departments is thus the major step to support decision-makers in planning emergency and long-term responses to climate change, employing urban design and planning to reduce community health vulnerability [64]. Fudge et al. argue that there is rarely a single solution to the complex problems that cities are facing, but rather engaging in transdisciplinary communication and collaboration may reduce adverse urban health outcomes [65]. The main barriers in planning in response to climate change are thus recognized in the lack of knowledge, financial and human resources, lack of accountability of local governments, and difficulties in engaging stakeholders [17].

#### 3.2.2. Assessment Process—Summary Analysis

- The tools aimed at understanding vulnerability to climate change risk are based on participatory approaches and stakeholder’s collaboration and aim at building adaptive capacity.
- The tools focused on health vulnerability aim at understanding health impacts according to population demographics and at increasing the collaboration of the health and planning sectors to support evidence-based decisions to increase resilience and reduce inequalities in health.
- The tools addressing the urban spaces focus on fostering the dialogue among different stakeholders and support planners in the development of resilient cities, through a network of safe, accessible and green public spaces.
- Although common patterns can be identified, none of the tools investigate the combination of health risk, climate change and population vulnerabilities in relation to public spaces.
- Breaking down siloed knowledge has been recognized as a major step to support long-term responses to climate change and the reduction in health vulnerability.

### 3.3. The Creation of Co-Benefits

Sub question 3: What co-benefits can result from the planning of healthy and inclusive public spaces?

Health co-benefits refer to the measures to address climate change that present positive social and health externalities. They are well documented and significantly outweigh the costs of implementing climate actions [13].

The quality of public spaces can generate significant social impacts that may be reflected in co-benefits for the community [66]. Identifying the solution with the greatest value, optimizing the use of resources, and assessing costs and benefits from a life-cycle perspective can foster social, environmental, and economic sustainability [66].

Identifying the needs to be satisfied does not constitute an unnecessary investment of time as, usually, design expenses are relatively modest compared to the costs of the construction and, much more so, the maintenance [66]. The latter is often the most expensive aspect, and it is thus necessary to evaluate the economic sustainability and feasibility of a project, providing design solutions that balance the valorization of the space and satisfaction of users’ needs, with the possibility of being adaptable to different contexts (Van Deurs, 2021). However, relatively few architects pay attention to the role of the environment in affecting people’s needs and behavior [66].

Well-designed and maintained public spaces can fulfil the needs of diverse communities, increasing land value and generating economic benefits for the city [18]. The financing of public spaces projects is strained by local government budgets, thus common solutions include partnerships with the private sector or philanthropic foundations [36]. They play an important role in financing health-related projects, looking at the connection between the environment and climate (Schuff & Spitzer, 2021).

The co-benefits of a project depend on the extent to which new green spaces or climate change mitigation and adaptation strategies improve people’s health [57]. The necessity of integrating health into policies can be supported through the estimation of the cost of action (new policies that address risks) and inaction (no policies) [53]. The latter includes expenditures to cover climate-change-related health impacts, described in Section 1 [53]. Challenges can arise in the collection and analysis of data, for example in the integration of climate and health data, depending on the context (neighborhood level, global south or global north, etc.) and the nature of the data (health diseases, pollution, etc.); in general, softer data are harder to obtain (Schuff & Spitzer, 2021).

The use of soft data allows to analyze human behavior in spaces and their perception can support the design phases in integrating different functions, in order to attract different people (Jens, 2021). In this way, it is also possible to make more evident the added value of a project and attract private investors, if it is aligned with their agenda, ensuring a better relationship between the private and public sectors (Schuff & Spitzer, 2021). 

Although methods to estimate economic benefits and costs of implementation are available in the literature, they do not often address the health co-benefits associated with climate change mitigation and adaptation strategies, e.g., increasing green space, providing shade, soft mobility solutions, and improved public transportation systems [34]. These solutions may provide both immediate and long-term health benefits, reduce greenhouse gas emissions, and result in significant economic returns [34]. 

#### The Creation of Co-Benefits—Summary Analysis

- Health, social, environmental, and economic co-benefits can result from public spaces planning strategies aimed at increasing the response to climate change impacts in cities.
- The use of data can support the design of public spaces in fulfilling the needs of different population groups and generate economic benefits.
- The analysis of the costs of action and inaction can promote the integration of health in public spaces and climate policies.
- Main gaps have been recognized in the collection of health data and in the assessment of health co-benefits of climate change mitigation and adaptation strategies in the literature.

### 3.4. Plan Healthy and Inclusive Public Spaces to Build Resilient and Liveable Cities

Research question: How to support the planning and design of healthy and inclusive public spaces, addressing both causes and effects of climate change, to build resilient and livable cities? 

Increasing access to high-quality public spaces for all urban residents can improve equity, promote inclusion, and thereby reduce social vulnerability by providing people with equal opportunities for development [49]. Furthermore, urban planning that considers environmental and human health does include a strong focus on the provision of green areas, children- and family-friendly spaces, community gardening, and blue and green infrastructures, contributing to increased air and water quality [67].

In the city of Copenhagen, playground areas are often designed to include recreational spaces also for adults, inviting people to be more active and promoting health for all ages (Van Deurs, 2021). The provision of public spaces should follow the demographics of the area to provide an adequate number of infrastructures for leisure and sports, and attractive places that can increase city-wide connectivity through basic mobility infrastructures, such as bike lanes and sidewalks, designed not only for young and healthy people, but also for the elderly and children (Van Deurs, 2021).

A city-wide strategy allows for visual and physical connections between streets and spaces and helps to make the design process easier and the planning project approval faster, helping the governments to reduce inequalities and reallocate benefits [28,36]. Architecture and planning can, therefore, be regarded as tools for addressing health and climate challenges at the local level, building healthy communities and increasing the quality of life for all [68].

Urban design strongly influences the quality, accessibility, access, sense of safety of a place, and determines how it is going to be used by different users [11]. At the design level, integrating health and planning in public spaces means considering how the physical environment influences health, through air and water quality, access to housing and transport systems, active mobility choices, and how microclimatic conditions, such as temperature, wind, sunlight, and shade, favor outdoor activities in public spaces [69] (Schuff & Spitzer, 2021).

It is necessary to understand the relationship to be fostered with the environment in relation to climate change: protection in different weather conditions, the perceptions of people about the outdoor space, and the influence on the use of the area can explain why some areas are more popular than others (Schuff & Spitzer, 2021). Understanding this relationship allows planners to have a more workable scale for tackling climate change and provide an offer for a diversity of users, through the variety of buildings and uses(Schuff & Spitzer, 2021). In Gehl’s professional practice, understanding people’s behavior and how it varies according to age and social status is crucial to this end: the firm developed several tools to support city officials in designing places for coexistence, avoiding exclusionary elements of architecture, for example, towards children or the homeless (Schuff & Spitzer, 2021). Therefore, the focus is not just on design, but on placing users at the core of the project and understanding how and when to involve them in the process (Schuff & Spitzer, 2021).

The architectural firm Henning Larsen integrates health and well-being, aligning the space with the users’ needs by testing the projects very early in the design phase, followed by monitoring, follow-up, and improvement phases (Jens, 2021). The use of sensors can provide an effective tool to check if the design intentions are met and to provide a basis for the discussion with the clients (Jens, 2021).

People are not only the victims, but also the drivers of climate change, hence they can act as agents in directing sustainable development by getting involved in participatory processes and response measures [70]. Breaking down knowledge silos and improving community participation at all levels of governance thus not only enables the social dimensions of climate change to be addressed, but can foster solutions for health and well-being, emergency preparedness, and disaster response [67]. Among public participation processes, placemaking enables architects and urban designers in the engagement of community in the co-creation of public spaces using local knowledge and building trust and a sense of belonging [18]. Placemaking is also focused on building-resilient infrastructures, such as nature-based solutions, that can mitigate climate change impacts and improve aesthetics, access, and connectivity to an area [18]. When is not possible to ensure the presence of spaces for climate mitigation and adaptation in the municipality areas, planners can find support in the local development plans to locate these areas in the private developments (Van Deurs, 2021).

Cost-effective and rapid-testing approaches to planning can also include tactical urbanism initiatives, where the community is directly involved in the co-generation of space [30]. These strategies can be used for pilot projects, before ensuring they are permanent and require substantial investment, but also for engaging with different stakeholders and the private sector (Carbajal & Mutai, 2021). 

#### Plan Healthy and Inclusive Public Spaces to Build Resilient and Livable Cities—Summary Analysis

- City-wide strategies for public spaces that tackle climate and health issues through the participation of the local communities and the different stakeholders can increase urban resilience and the sense of belonging, creating livable cities.
- A network of high-quality and green public spaces can promote social inclusion, reduce vulnerabilities and help to address climate change challenges.
- Understanding the perception of the users about the space (through sensors and participation processes) allows the designers to adapt the project to the population’s needs.

## 4. Discussion

In the following section, key findings from both the literature and interviews will be discussed, answering the research questions.

The planning and design of public spaces that incorporate solutions for climate change mitigation and adaptation can result in multiple co-benefits for human health in cities. The provision of green areas contributes to improved air quality, reduces heat island effects, and provides shade from the sun, increasing both mental and physical health. 

The design of public spaces should guarantee the satisfaction of the needs of different population groups, particularly the most vulnerable, contributing to the reduction in social inequalities. Increasing the provision, access and accessibility of public spaces can strengthen social relations and a sense of community. In addition, connectivity through public transport routes, bicycle and pedestrian paths, and walkable neighborhoods stimulate physical activity, with a positive health impact, while lowering GHGs emissions.

The analysis shows that the main challenges in addressing the interlinkages between climate change and health in planning public spaces reside in the limited capacity across public sectors and policymakers, gaps in cross-sectoral knowledge and communication between health experts and planners, and shortage of human and economic resources. Moreover, the social impacts of climate change and the limited inclusion of health considerations in urban-level assessment methods and tools need to be addressed.

During the research, several tools designed to inform solutions to implement public spaces, reduce climate change impacts, or address vulnerabilities at the urban level were analyzed. However, a comprehensive tool for evaluating simultaneously the interlinkages between climate and health, social, environmental, and economic vulnerabilities, and the quality of the urban spaces at the local level has not been found, as fully addressed, in the literature.

The literature review and interviews also identified health data collection as one of the major challenges in assessing the existing situation, especially in the global south and informal settlements, while climate data are considered easier to obtain. There is consensus that, among the first data to be collected, there are demographics, in order to identify population groups, their age, economic status, social cohesion, and exposure to risk.

The most common methods employed in the selected tools are the mapping and the involvement of as many stakeholders as possible in the process, representing different interests, through meetings and workshops. Mapping allows to understand the distribution of hazards, risks and population needs, and then to identify the most appropriate actions to be taken in different areas.

The best strategies to reduce the impacts of climate change were recognized in early warning systems, knowledge sharing, and information to the population, along with the monitoring and implementation of the plans. A planning project that considers all these aspects together can therefore provide several co-benefits, for health, economy, climate, and community cohesion.

The planning of public spaces aimed at meeting different needs can enhance social inclusion and community cohesion. Furthermore, benefits for health can arise from increased physical activity, reducing health risks, such as obesity and cardiovascular disease, as well as by improved air and water quality and microclimatic conditions, for example, by reducing the spread of vector-borne diseases, noise levels, and heat island effect. Lowering stress levels and increasing safety, e.g., by disaster risk reduction and road accident prevention through safe slow mobility routes, not only can improve physical, but also mental health. Numerous economic co-benefits can result from the design of resilient public spaces, such as the support of local economies, but also the prevention of hazards and damage to the built environment, the reduction in mortality and health diseases, and the reduction in maintenance costs of public areas.

**Figure 3:**
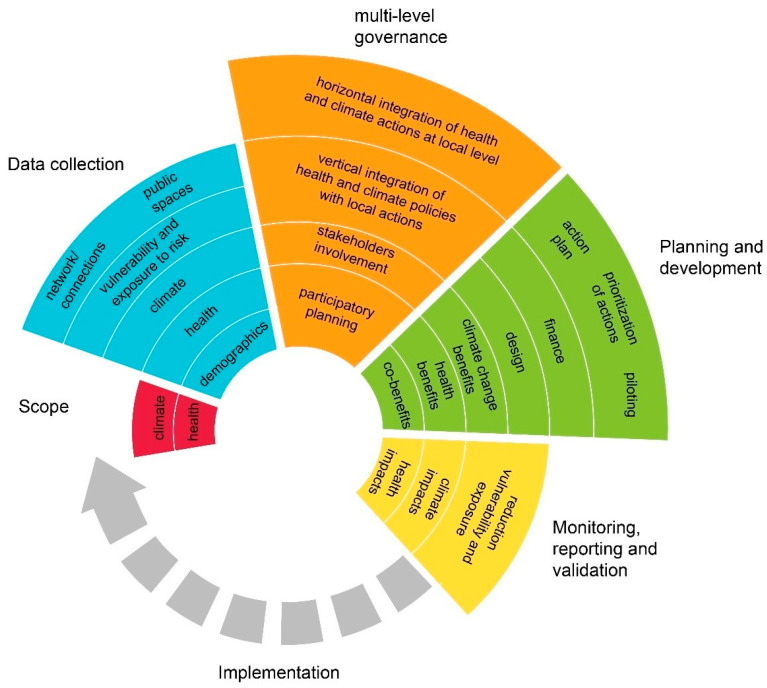
Preliminary set of recommendations for a planning process.

To develop a preliminary set of recommendations for a planning process of resilient, livable, and healthy public spaces, several assessment tools were selected and analyzed in Section 3 and a general process was recognized and is implemented in Figure 3.

After clarifying the scope of the assessment, the data collection phase should include indicators about demographics (age, economic status, gender, education, households, ethnicity, population density, population growth, etc.), present and future health conditions (physical and mental), climate change present and future hazards, vulnerability and exposure to risk, and adaptive capacity analysis. Current strategies, policies, and measures regarding public spaces, together with the assessment of the physical features of the urban spaces (provision, distribution, access, accessibility, and quality) and their network and connections need to be addressed.

Health and climate actions should be integrated at the local level and respond to national and global policies. A transparent and participatory decision-making process ensures the inclusion and empowerment of often marginalized populations. Stakeholders from national and local government, policymakers, public health professionals, urban dwellers, community leaders and members, organizations, experts and representatives from gender groups, youth and human rights council, persons with disabilities, elderly and representatives from women and children, private sector, academia, NGOs, migrants, and refugees, need to be involved in the process. The goal is to promote knowledge sharing, create interest and awareness, and build capacity through workshops and meetings.

A shared planning proposal, guided by urban planners, allows to evaluate the effectiveness of mitigation and adaptation measures, climate and health benefits using short- and long-term scenarios, design proposals and financing options through actions plans and piloting of temporary testing solutions.

Monitoring health (pollution, green areas, air and water quality, the burden of diseases, etc.) and climate impacts (heat islands, rainfalls, water management, etc.), together with the reduction in vulnerability and exposure to risk can lead to understand the actions for further implementation.

The issues addressed are highly complex both in understanding the impacts and causes and in defining solutions and are accompanied by a high degree of uncertainty. As the degree of uncertainty decreases, preliminary results are obtained that enable the process to be adjusted over time. Resilience can thus tackle both the causes and effects of major global challenges, aiming at establishing dynamically adaptive processes. The process can support local governments, policymakers, community leaders, research institutions, and private sector bodies as well as architecture and engineering professionals in making informed decisions in planning public spaces that address climate change impacts and promote health. It might be implemented, with possible contributions from health and planning professionals, and further developed as a tool, to be tailored to specific areas (e.g., according to climate, geography, economic status, etc.) or types of public spaces (indoor, outdoor, streets, plazas, etc.).

## 5. Conclusions

The research shows that, although planning has an important role in promoting health, the latter is not sufficiently considered in the public space planning process and therefore requires more attention.

Increasing the provision, access, and accessibility of a network of public spaces can generate multiple co-benefits for health, reducing the impacts of climate change, lowering vulnerabilities and inequalities, enabling the development of resilient cities.

To address the major research gaps, it is necessary to increase the capacity of the public sector, promote knowledge sharing between the planning and health sectors, and provide tools for the collection and analysis of health data. To this end, a process was suggested for the integration of different competencies and the involvement of different stakeholders in a participatory process with the aim of developing a tool for a comprehensive assessment of the interlinkages between climate and health, social, environmental, and economic vulnerabilities, and the quality of the urban spaces.

To develop a tool that can be used by practitioners, further research is thus needed to define a list of indicators for evaluation and a set of best practices based on local context, including considerations of geography, microclimate, and socioeconomic conditions. The concept of resilience needs to be operationalized in order to enhance the potential co-benefits for health and climate change, addressing impacts and risks as part of an integrated system when planning public spaces.

Among the main limitations of this research is the absence of a case study, specific typology of space, or geographical area. The purpose was rather to provide general guidelines for addressing the most common challenges, knowledge gaps, and methods in planning resilient and livable public spaces. Therefore, potential design solutions were not analyzed as they would need to be tailored to the local context and could not be generalized.

## Data Availability

Not applicable.
